# Comparison of anatomical-based vs. nTMS-based risk stratification model for predicting postoperative motor outcome and extent of resection in brain tumor surgery

**DOI:** 10.1016/j.nicl.2023.103436

**Published:** 2023-05-16

**Authors:** Meltem Ivren, Ulrike Grittner, Rutvik Khakhar, Francesco Belotti, Heike Schneider, Paul Pöser, Federico D'Agata, Giannantonio Spena, Peter Vajkoczy, Thomas Picht, Tizian Rosenstock

**Affiliations:** a Department of Neurosurgery, Charité –Universitätsmedizin Berlin, Corporate Member of Freie Universität Berlin, Humboldt-Universität zu Berlin, and Berlin Institute of Health, Charitéplatz 1, 10117 Berlin, Germany; b Department of Neurosurgery, Heidelberg University Hospital, Im Neuenheimer Feld 400, 69120 Heidelberg, Germany; c Institute of Biometry and Clinical Epidemiology, Charité –Universitätsmedizin Berlin, Corporate Member of Freie Universität Berlin, Humboldt-Universität zu Berlin, and Berlin Institute of Health, Charitéplatz 1, 10117 Berlin, Germany; d Neurosurgery Unit, Spedali Civili di Brescia Hospital, 25123 Brescia, Italy; e Department of Neuroscience, University of Turin, Via Verdi 8, 10124 Turin, Italy; f Cluster of Excellence: “Matters of Activity. Image Space Material,” Humboldt University, Unter den Linden 6, 10099 Berlin, Germany; g Berlin Institute of Health at Charité – Universitätsmedizin Berlin, BIH Biomedical Innovation Academy, BIH Charité Digital Clinician Scientist Program, Charitéplatz 1, 10117 Berlin, Germany

**Keywords:** Motor outcome, Brain tumor surgery, Navigated transcranial magnetic stimulation (nTMS), diffusion tensor imaging (DTI), Glioma, Extent of resection

## Abstract

**Background:**

Two statistical models have been established to evaluate characteristics associated with postoperative motor outcome in patients with glioma associated to the motor cortex (M1) or the corticospinal tract (CST). One model is based on a clinicoradiological prognostic sum score (PrS) while the other one relies on navigated transcranial magnetic stimulation (nTMS) and diffusion-tensor-imaging (DTI) tractography. The objective was to compare the models regarding their prognostic value for postoperative motor outcome and extent of resection (EOR) with the aim of developing a combined, improved model.

**Methods:**

We retrospectively analyzed a consecutive prospective cohort of patients who underwent resection for motor associated glioma between 2008 and 2020, and received a preoperative nTMS motor mapping with nTMS-based diffusion tensor imaging tractography. The primary outcomes were the EOR and the motor outcome (on the day of discharge and 3 months postoperatively according to the British Medical Research Council (BMRC) grading). For the nTMS model, the infiltration of M1, tumor-tract distance (TTD), resting motor threshold (RMT) and fractional anisotropy (FA) were assesed. For the PrS score (ranging from 1 to 8, lower scores indicating a higher risk), we assessed tumor margins, volume, presence of cysts, contrast agent enhancement, MRI index (grading white matter infiltration), preoperative seizures or sensorimotor deficits.

**Results:**

Two hundred and three patients with a median age of 50 years (range: 20–81 years) were analyzed of whom 145 patients (71.4%) received a GTR. The rate of transient new motor deficits was 24.1% and of permanent new motor deficits 18.8%. The nTMS model demonstrated a good discrimination ability for the short-term motor outcome at day 7 of discharge (AUC = 0.79, 95 %CI: 0.72–0.86) and the long-term motor outcome after 3 months (AUC = 0.79, 95 %CI: 0.71–0.87). The PrS score was not capable to predict the postoperative motor outcome in this cohort but was moderately associated with the EOR (AUC = 0.64; CI 0.55–0.72). An improved, combined model was calculated to predict the EOR more accurately (AUC = 0.74, 95 %CI: 0.65–0.83).

**Conclusion:**

The nTMS model was superior to the clinicoradiological PrS model for potentially predicting the motor outcome. A combined, improved model was calculated to estimate the EOR. Thus, patient counseling and surgical planning in patients with motor-associated tumors should be performed using functional nTMS data combined with tractography.

## Highlights

• The nTMS model with its functional data and nTMS-based tractography was superior to the clinicoradiological PrS model for predicting motor outcome in motor eloquent brain tumor resection.
• The cortical nTMS mapping, the tumor-tract distance, the white matter integrity and the cortical excitability were integrated into a regression tree analysis to predict the short- and longterm motor outcome.
• A combined model has been developed to predict the EOR most accurately using parameters from both models.
• Patients with motor-eloquent brain tumors should be treated in large centers with expertise in nTMS and tractography to achieve the optimal functional outcome and extent of resection.

## 1 Introduction

Although malignant primary brain tumors are rare, with an incidence of 7 per 100.000, they represent a drastic event for the patient and require demanding, individualized treatment concepts (Ostrom et al., 2020). Surgical resection is the first choice for the treatment of glioma, while a complete tumor removal/gross total resection (GTR) is correlated with higher progression-free and overall survival rates (Almenawer et al., 2015, Li et al., 2016, Molinaro et al., 2020, Wykes et al., 2021). Some studies have explored the concept of supramarginal resection (tumor resection beyond the contrast-enhanced region on MRI), but this has to be balanced against the risk of affecting eloquent brain regions, resulting in permanent neurologic deficits (Wang et al., 2021). Rolandic or insular tumors, for example, carry the risk of inducing a new postoperative motor deficit which can result from direct injury to the motor cortex/pyramidal tract or from ischemia. Perioperative motor deficits have not only been reported to negatively affect overall survival and quality of life despite standard surgical and adjuvant treatment, but also to be responsible for patients being less likely to receive radio- or chemotherapy in the first place (Gulati et al., 2011, Jakola et al., 2011b, McGirt et al., 2009, Rahman et al., 2017).

While characteristics defining survival have been widely explored in the past, literature on predictive aspects of functional outcome in rolandic tumor surgery is relatively sparse. To our knowledge, there are two proposed prognostic models for predicting postoperative motor outcome to determine the individual treatment strategy. One regression model uses functional data from navigated transcranial magnetic stimulation (nTMS) and data from diffusion tensor imaging (DTI) to estimate the risk for a new motor deficit/worsened motor status on the day of discharge and after 3 months (Rosenstock et al., 2017b, Rosenstock et al., 2021a). Another model calculates a sum score using clinical and anatomical (MRI-based) parameters to predict the postoperative functional outcome (according to the modified Rankin Scale [mRS]) and the extent of resection (EOR) (Spena et al., 2018). The aim of this study was to compare these two models using the same prospective dataset, identify unique strengths and weaknesses, and potentially develop an improved model. We hypothesize that combining the models will result in higher predictive accuracy and better discrimination ability.

## 2 Material and methods

### 2.1 Study design

This retrospective study was carried out in accordance with the Ethics Commission of the Charité – Universitätsmedizin Berlin (EA1/016/19), the STROBE-Guidelines (von Elm et al., 2014), and the Declaration of Helsinki. All patients who underwent surgery for (presumed) motor-eloquent glioma between 2008 and 2020 were included in a prospective dataset. Both prognostic models for predicting postoperative motor outcome were applied to this dataset to evaluate their predictive power, whereas the nTMS model is part of the clinical routine and was applied prospectively accordingly. Included patients suffered from neuroepithelial tumors closely related to the motor cortex and/or the corticospinal tract (CST), consistent with the inclusion criteria of the initial publications (Rosenstock et al., 2021a, Spena et al., 2018). Other histologies were considered as exclusion criteria. Muscle strength was documented preoperatively, at the day of discharge and three months postoperatively according to the British Medical Research Council (BMRC) grading of 6 grades (0–5), where 0 means plegia and 5 means fully preserved strength(James, 2007). If the postoperative BMRC score was worse than the preoperative score, this was considered as motor deterioration. Collected data included patient sex, age, Karnofsky Performance Scale (KPS), preoperative seizures, preoperative sensory and/or motor deficits, tumor histology according to the WHO grading (Perry and Wesseling, 2016), as well as the below mentioned parameters of both models. The KPS is a score ranging from 0 to 100% that indicates the degree of independence in daily living, with 100% indicating complete independence and lower scores indicating the degree of need for assistance (Schag et al., 1984).

### 2.2 Introduction of the nTMS model

**Fig. 1:**
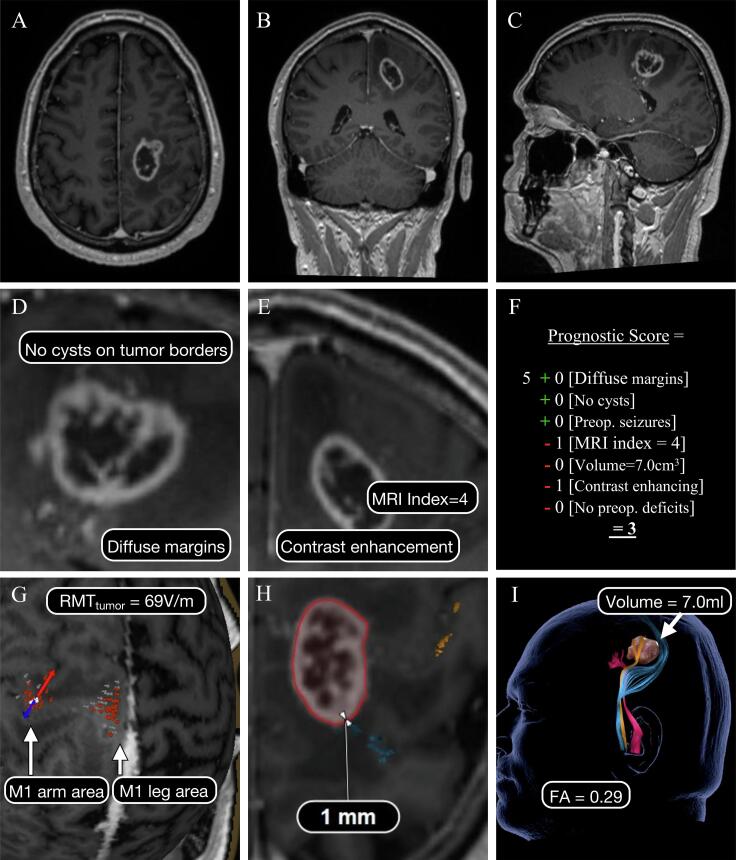
Application of the PrS and nTMS model to an exemplary case. MRI scan (T1 with contrast agent) show a left central tumor (**A-C**) of a 62-year-old man. Diffuse margins of the contrast-enhancing tumor, an MRI index of 4 and no peritumoral cysts (**D, E**) equate to a a PrS of 3, meaning 8% chance for GTR and 50% risk of neurologic deterioration (**F**). The tumor is located subcortically under the motor cortex. Thus, the nTMS mapping did not demonstrate a tumorous infiltration of the motor cortex (**G**) but an impaired neurophysiological excitability (RMT_tumor_ = 69 V/m; RMT_ratio_ = 82%). The colored spots mark stimulation sites where motor-evoked potentials with different amplitudes were elicited. These functional areas were used as region of interests for tractography to somatotopically visualize the CST for the lower extremity (blue), upper extremity (yellow), and corticonuclear tracts (pink) (**H, I**). Due to compromised white matter integrity (FA = 0.29) and TTD of 1 mm, there is a 30.3% risk for transient and 15.6% risk for permanent motor deterioration. A GTR could be achieved but the patient suffered a 2/5 hemiparesis on discharge which recovered to BMRC grade 4/5 after 3 months.

Both models, their concepts and their endpoints - as originally designed by the authors - are contrasted in Table 1 and visualized in Fig. 1. For easier readability, the models will be referred to as the nTMS model and the PrS (Prognostic sum score) model.

**Table 1:** Comparison of the two models.

	nTMS model	PrS model^3^
Model Characteristics	1.) Functional data from nTMS	1.) Clinical data
	2.) Diffusion data from DTI tractography	2.) MRI-based anatomical characteristics
Output	Risk for postoperatively deteriorated motor status (BMRC score)	Probability of
	1.) at day of discharge and	1.) favorable neurologic outcome (mRS improved or unvaried) after 6 months
	2.) after 3 months	2.) GTR
Input	nTMS data: Infiltration of motor cortex; RMT ratio	Tumor margins; cysts; seizures at onset; MRI index; tumor volume; contrast enhancement; paresis/dysesthesia at onset
	DTI data: Distance tumor-CST; FA	
Statistical Methods	Multiple ordinal regession model (n = 113)^1^ and validation with regression tree analysis (n = 165)^2^	PrS = 5 + sharp margins + cyst present + seizure at onset – paresis – MRI index > 2 – volume > 80 cm3 – contrast enhancement
	Risk short-term motor deterioration:	Relative frequencies, depending on subgroups of the sum score
	I) 1.6%	PrS 1–3: 8% GTR; 50% favorable neurological outcome
	II) 19.5%	PrS 4: 58% GTR; 83% favorable neurological outcome
	IIIA) 30.3%	PrS 5: 80% GTR; 100% favorable neurological outcome
	IIIB) 58.6%	PrS 6–7: 100% GTR; 100% favorable neurological outcome
	Risk long-term motor deterioration:	n = 48
	I) 0%	
	II) 6.5%	
	III) 15.6%	
Tumor Location	1) Compressing or infiltrating motor cortex	Infiltration or close vicinity to precentral or postcentral gyrus or CST
	2) Close relationship to CST	
Tumor Histology	Glioma	Glioma
Recurrent Tumors	Included	Excluded
Definition of EOR	GTR: no residual contrast-enhancing tissue on T1-weighted images and no residual hyperintense tissue on FLAIR images of non-enhancing tumors	GTR: ≥ 95% resected;
	STR: residue ≤ 15 ml	STR: 85–95% resected;
	PR: residue > 15 ml	PR: < 85% resected

**1** = (Rosenstock et al., 2017b). **2** = (Rosenstock et al., 2021a). **3** = (Spena et al., 2018). **nTMS** = Navigated transcranial magnetic stimulation. **PrS** = Prognostic sum score. **DTI** = Diffusion tensor imaging. **BMRC** = British Medical Research Council. **mRS** = Modified Rankin Scale. **GTR** = Gross total resection. **RMT** = Resting motor threshold. **CST** = Corticospinal tract. **EOR** = Extent of resection. **STR** = Subtotal resection. **PR** = Partial resection.

The nTMS model is based on navigated transcranial magnetic stimulation, a noninvasive method for mapping cortical motor function in relation to the tumor site by using electromagnetic induction as previously described elsewhere (Levy et al., 1991, Picht et al., 2009). Thus, tumor-induced plasticity as well as the neurophysiology of the motor system was assessed for all patients. Cortical excitability is analyzed via the Resting Motor Threshold (RMT), defined as the minimum stimulation intensity needed to evoke a motor potential of 50 µV in at least five out of ten stimulations. The RMT_ratio_ (=RMT_tumor_ of the tumorous hemisphere/RMT_healthy_ of the contralateral hemisphere) is used to assess the excitability level between the hemispheres. The minimum distance between the tumor and the CST is determined through nTMS-based tractography (explained in detail below) (Frey et al., 2012, Rosenstock et al., 2017a). A cutoff value of 8 mm was used for the tumor-tract distance (TTD) because no patient had a new postoperative motor deficit when the TTD was >8 mm (Rosenstock et al., 2017b).

In the validation of the initial model, the relevance of the white matter tract integrity (represented by the fractional anisotropy (FA) value) was shown (Rosenstock et al., 2021a). Therefore, we assessed the following risk parameters for an association with postoperative motor outcome:

- nTMS-verfied tumor infiltration of the primary motor cortex.

- Minimum distance between tumor and CST.

- RMT_ratio_, RMT_tumor_, RMT_healthy_ (measured at a peeling depth of 22.5 mm).

- FA.

Prediction of the EOR was not part of the nTMS model.

### 2.3 Introduction of the PrS model

The PrS model aims to estimate the probability of achieving a GTR as well as favorable neurological outcome based on a clinicoradiological sum score which was calculated retrospectively (Spena et al., 2018). In contrast to the nTMS model, the PrS model used the mRS, with a maintained or improved score at 6 months defined as a “favorable outcome”. In contrast to the original PrS model, we assessed the EOR by the absolute residual tumor volume, following the EANO guidelines (Weller et al., 2021). Favorable parameters (sharp margins; cystic components; preoperative seizure) increase and unfavorable parameters (contrast agent enhancement, tumor volume, MRI index (Spena et al., 2013), preoperative sensorimotor deficit) decrease the sum score. After subgrouping according to the original model, higher scores indicate a higher likelihood of achieving a GTR and a favorable outcome. The exact calculation of the score is presented in Table 1 and exemplarily described in Fig. 1. The chance of a favorable outcome and a GTR results from the relative frequencies of the subgroups of the initial model. Please note that cystic components, as specified by the authors, only contain cysts on the tumor borders (Spena et al., 2018). The MRI index grades subcortical white matter infiltration as follows: An index of 1 describes the limitation of the tumor to solely one gyrus; an index of 2 describes tumors that extend from one gyrus to the white matter and/or an adjacent gyrus; an index of 3 defines lesions that infiltrate up to three gyri with extension to white matter tracts; an index of 4 classifies subcortical lesions, and an index of 5 is assigned to lobar tumors. (Spena et al., 2013, Spena et al., 2018).

### 2.4 MRI data Attainment, nTMS motor mapping & tractography

MR imaging was carried out on a 3-T Skyra scanner (Siemens, Erlangen, Germany) as described previously in detail (Rosenstock et al., 2021a). The T1 sequence with a slice thickness of 1 mm was used for the nTMS mapping (eXimia, Nexstim Oy) to detect tumorous infiltration of the motor cortex and to identify specific seed regions for the nTMS-based tractography of the CST (Fig. 1) (Rosenstock et al., 2017a). A maximum registration error of less than 2 mm was allowed for neuronavigation. The diffusion tensor imaging was obtained with single-shot echo-planar sequences along 60 different geometric directions at a b-value of 1300 s/mm^2^) with a resolution of 2 mm.

Technical details were described in detail earlier (Rosenstock et al., 2017b). Briefly, the presumed hand and leg areas of the motor cortex are stimulated. Motor evoked potentials (MEP) are then derived for the abductor pollicis brevis, first dorsal interosseus, tibialis anterior, and extensor hallucis longus muscles by electromyogram so that both the upper and lower extremities are represented. After the motor cortex is roughly identified, the so-called “hot spot,” i.e., the site that responds with the highest MEP amplitude to stimulation, is used to determine the resting motor threshold or RMT (minimum intensity required to elicit an MEP of 50 µV for at least five out of ten stimulations). The actual mapping is then performed at an intensity of 105% of the RMT for the upper limb and 130% of the RMT for the lower limb, where the 8-shaped coil ensures specific mapping with focal stimulation. The positive stimulation points are then transferred to the fiber tracking software (iPlan 3.0) - forming one of two regions of interest (ROI) for our fiber tracking (each point with a radius of 3 mm). We chose the anterolateral part of the ipsilateral pons (color-coded FA map) as the second ROI (Rosenstock et al., 2017a).

The area of visualized stimulation-positive points on the 3D-reconstructed T1 sequence (Fig. 1G) was defined as the motor cortex. Visible tumor tissue in close proximity to this area/inside the motor cortex was considered infiltration of the motor cortex, which was only assessed dichotomously and not quantified. For nonenhancing tumors, FLAIR images were visually assessed in comparison with the T1 images.

All images were analyzed by an interdisciplinary board of neuroradiologists and neurosurgeons, whereas the postoperative MRI was performed ≤ 48h after surgery to assess residual tumor volume using the T1 sequence with contrast agent for enhancing tumors or the FLAIR (T2 inversion recovery fast spin echo) sequence otherwise. Tumor volumetry and tractography of the CST were performed, and the tumor-tract distance (TTD) as well as the average FA value of the tracts were measured with our planning software (BrainLab Elements, Brainlab AG, Munich, Germany). Tumor volumetry and measurement of TTD (based on axial slices) were performed by the investigator, whereas examiner independence had already been proven in advance (Rosenstock et al., 2017a). Due to the retrospective analysis, FA values could not be obtained in 19 cases.

### 2.5 Surgery

Tumor resections in motor eloquent regions were performed only by neurosurgeons with extensive experience in the field of neuro-oncologic surgery. Intraoperative neuromonitoring (IOM) such as transcranial motor evoked potentials and/or subcortical stimulation via the surgical aspirator took place if the distance of the tumor to the pyramidal tract was less than 1 cm (Rosenstock et al., 2021b). No awake surgeries were performed. If achievable, the surgical team aimed for GTR, choosing a subcortical stimulation threshold between 2 and 5 mA depending on the chance for total resection and the risk profile for motor deterioration. Resection was interrupted if the MEP amplitude decreased over 50%. If the potentials did not recover after a five-minute interval of intraoperative irrigation and monitoring of vital parameters, resection was terminated.

### 2.6 Statistical analysis

To validate the models and, eventually, to calculate new combined models with optimized accuracy and discrimination ability, we proceeded as follows: 1.) bivariate binary logistic regression of the models' parameters with the motor outcome at days of discharge, after 3 months and the EOR, 2.) comparison of the accuracy of the original models using ROC analysis, 3.) calculating new models based on regression tree analyses with all outcome-associated parameters identified by the bivariate binary logistic regression.

Data was statistically analyzed with SPSS (IBM SPSS Statistics version 27, IBM corp.) and R (R Core Team, 2021). Main characteristics of study patients are decribed in Table 2 using absolute and relative frequencies as well as mean, standard deviation, median, interquartile range and range depending on the scaling of the variables. EOR was dichotomously divided into GTR vs. non-GTR. The postoperative motor outcome was dichotomously divided into worsened or not worsened (equal/improved motor status) as measured by the BMRC score. Every component of the PrS and the nTMS model score were evaluated for association with the motor outcome and EOR using separate binary logistic regression analysis (Table 3). Table 3 provides descriptive measures (absolute and relative frequencies) of the three outcomes in subgroups of parameters and odds ratios (OR) with 95% confidence intervals from the separate binary logistic regression models. Regression tree analysis was used for classification of the three outcomes depending on nTMS measures. For postoperative motor outcome the previously published regression trees were fitted to the larger study population here. The R package rpart (Recursive Partitioning and Regression Trees) was used for the regression tree analyses. For EOR a new regression tree model was calculated using 10-fold cross validation, a complexity parameter of 0.03, a minimum number of observations in a node of 30, a minimal number of observations in a terminal leaf of 10, splitting index: Gini coefficient and a depth of 3 nodes. Discrimination ability of the models was evaluated by calculating AUC (area under the curve) and 95% CI for probabilities coming from the regression trees for nTMS measures as well as for the PrS Score.

**Table 2:** Patient characteristics.

n	203
**Sex**	
Male	128 (63.1%)
Female	75 (36.9%)
**Age in years,** mean (SD) [range]	50 (15) [20–81]
**KPS**	
≤70%	30 (14.8%)
80%	21 (10.3%)
90%	72 (35.5%)
100%	80 (39.4%)
**Affected hemisphere**	
Right	110 (54.2%)
Left	92 (45.3%)
Bilateral	1 (0.5%)
**Histology**	
HGG	173 (85.2%)
LGG	30 (14.8%)
**Recurrency**	
Primary tumor	149 (73.4%)
Recurrent tumor	54 (26.6%)
**Tumor volume in ml,** median (IQR) [range]	27 (13–54) [0.2–224]
**Preoperative BMRC grade**	
BMRC ≤ 3	19 (9.4%)
BMRC 4	54 (26.6%)
BMRC 5	130 (64.0%)
**EOR**	
GTR	145 (71.4%)
STR	49 (24.1%)
PR	9 (4.4%)
**Postoperative motor outcome**	
Transient deterioration (day of discharge)	49 (24.1%)
Permanent deterioration (3 months)	36 (18.8%)^1^

**KPS** = Karnofsky performance status, **HGG** = high grade glioma, **LGG** = low grade glioma, **BMRC** = British Medical Research Council, **GTR/STR/PR** = Gross total/Subtotal/Partial resection, **SD** = Standard deviation. **1** = Follow-up data 3 months postoperatively were available for 184 patients.

**Table 3:** Association between parameters of nTMS/PrS model with EOR and postoperative motor outcome.

	EOR				Postoperative motor outcome (day of discharge)				Postoperative motor outcome (after 3 months)			
	total	no GTR	OR (95 %CI)	p	total	worsening	OR (95 %CI)	p	total	worsening	OR (95 %CI)	p
n	203	58 (28.6%)			203	49 (24.1%)			192	36 (18.8%)		
**Parameter of nTMS model**												
**M1 infiltration**				<0.001				0.007				0.006
Yes	39	21 (53.8%)	4.01 (1.93–8.30)		39	16 (41.0%)	2.76 (1.31–5.81)		37	13 (35.1%)	3.11 (1.39–6.97)	
No	164	37 (22.6%)	1		164	33 (20.1%)	1		155	23 (14.8%)	1	
**TTD**				<0.001				<0.001				<0.001
≤ 8 mm	129	49 (38.0%)	4.42 (2.02–9.67)		129	47 (36.4%)	20.63 (4.84–87.97)		122	36 (29.5%)	45.97 (6.25–586.51)	
> 8 mm	74	9 (12.2%)	1		74	2 (2.7%)	1		70	0	1	
**RMT_tumor_**				0.116				0.058				0.027
< 71 V/m	119	29 (24.4%)	1		119	23 (19.3%)	1		112	15 (13.4%)	1	
≥ 71 V/m	84	29 (34.5%)	1.64 (0.89–3.03)		84	26 (31.0%)	1.87 (0.98–3.58)		80	21 (26.3%)	2.30(1.10–4.81)	
**RMT ratio**												
<90%	76	23 (30.3%)	1.33 (0.62–2.85)	0.714	76	21 (27.6%)	2.53 (1.03–6.21)	0.06	71	18 (25.4%)	3.67 (1.27–10.60)	0.055
90%-110%	61	15 (24.6%)	1		61	8 (13.1%)	1		59	5 (8.5%)	1	
>110%	66	20 (30.3%)	1.33 (0.61–2.92)		66	20 (30.03%)	2.88 (1.16–7.16)		62	13 (21.0%)	2.87 (0.95–8.62)	
**FA^1^**				<0.001				0.002				<0.001
< 0.47	79	34 (43.0%)	4.91 (2.40–10.07)		79	29 (36.7%)	3.00(1.50–6.00)		72	23 (31.9%)	7.43(2.84–19.47)	
≥ 0.47	105	14 (13.3%)	1		105	17 (16.2%)	1		101	6 (5.9%)	1	

Parameters of PrS model												
**Tumor margins**				0.133				0.901				0.241
Sharp	101	24 (23.8%)	1		101	24 (23.8%)	1		97	15 (15.5%)	1	
Diffuse	102	34 (33.3%)	1.60 (0.87–2.97)		102	25 (24.5%)	1.04 (0.55–1.98)		95	21 (22.1%)	1.55 (0.75–3.23)	
**Cyst**				0.009				0.907				0.203
Yes	51	22 (43.1%)	2.44 (1.25–4.77)		51	12 (23.5%)	0.96 (0.45–2.02)		48	12 (25.0%)	1.67 (0.76–3.66)	
No	152	36 (23.7%)	1		152	37 (24.3%)	1		144	24 (16.7%)	1	
**Preop. Epilepsy**				0.556				0.13				0.869
No	81	25 (30.9%)	1.20 (0.65–2.23)		81	15 (18.5%)	0.59 (0.30–1.17)		77	14 (18.2%)	0.94 (0.45–1.97)	
Yes	122	33 (27.0%)	1		122	34 (27.9%)	1		115	22 (19.1%)	1	
**MRI index**				0.007				0.56				0.142
3-5	151	51 (33.8%)	3.28 (1.38–7.79)		151	38 (25.2%)	1.25 (0.59–2.68)		141	30 (21.3%)	2.03 (0.79–5.20)	
1–2	52	8 (13.5%)	1		52	11 (21.2%)	1		51	6 (11.8%)	1	
**Tumor volume**				0.042				0.44				0.684
>80 ml	32	14 (43.8%)	2.25 (1.03–4.89)		32	6 (18.6%)	0.69 (0.27–1.78)		31	5 (16.1%)	0.81 (0.29–2.27)	
≤80 ml	171	44 (25.7%)	1		171	43 (25.1%)	1		161	31 (19.3%)	1	
**Contrast enhancement**				0.657				0.651				0.592
Yes	146	43 (29.5%)	1.17 (0.59–2.33)		146	34 (23.3%)	0.85 (0.42–1.72)		137	27 (19.7%)	1.26 (0.55–2.88)	
No	57	15 (26.3%)	1		57	15 (26.3%)	1		55	9 (16.4%)	1	
**Preop. paresis dysesthesia**				0.026				1				0.262
Yes	87	32 (36.8%)	2.01 (1.09–3.73)		87	21 (24.1%)	1.0 (0.52–1.92)		80	18 (22.5%)	1.52 (0.73–3.14)	
No	116	26 (22.4%)	1		116	28 (24.1%)	2.0 1		112	18 (16.1%)	1	

Testing for group differences was performed with bivariate binary logistic regression. **GTR** = gross total resection. **M1** = motor cortex. **TTD** = tumor-tract distance. **RMT** = resting motor threshold. **FA** = fractional anisotropy. **1** = Due to missing FA values in 19 patients, only 184 patients were analyzed.

## 3 Results

### 3.1 Patient sample

The patient characteristics are summarized in Table 2, their association to the postoperative motor outcome in Suppl. data 1**.** The prospective data set included 203 patients with a mean age of 50 years (SD: 15 years, range 20–81 years) and a median KPS of 90% (range 40%–100%). Eleven patients were lost to follow-up: five patients (2.5%) continued their treatment elsewhere; four patients (2.0%) deceased; one patient (0.5%) experienced secondary motor worsening due to a satellite lesion, therefore we excluded the follow-up BMRC score. In one case (0.5%), the reason for loss of follow-up is unknown. The rate of new postoperative motor deficits was 24.1% at the day of discharge and 18.8% after three months. A GTR was achieved in 145 patients (71.4%), a subtotal resection in 49 (24.1%) and a partial resection in 9 cases (4.4%).

Patients with a recurrent tumor had a higher risk to suffer a new permanent postoperative motor deficit (14 out of 48, 29.2%) compared to those with primary tumors (22 out of 144, 15.3%, p = 0.036) (Suppl. data 1). This association was similar but not as pronounced for the short-term postoperative motor outcome (31.5% vs. 21.5%, p = 0.143, Suppl. data 1). No substantial association between recurrent tumors and EOR could be identified.

### 3.2 Validation of the nTMS model

**Supplementary figure 1:**
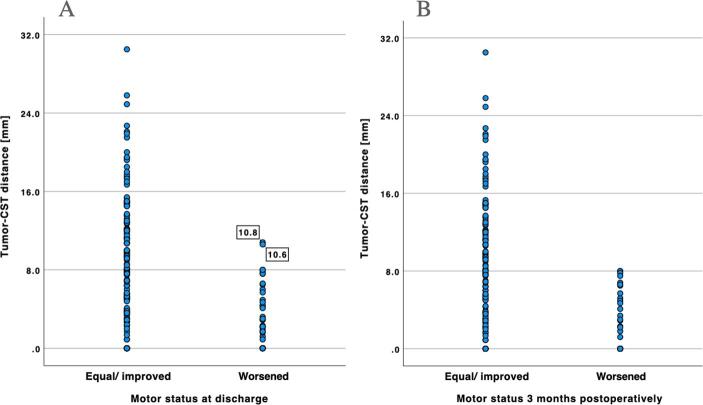


The parameters of the nTMS model and their bivariate association with postoperative motor outcome and EOR are shown in Table 3. The preoperative DTI sequence was not available in 19 patients, so these cases had to be excluded for the nTMS model. Tumor infiltration of the motor cortex was evident in 39 cases (19.2%), whose likelihood of new postoperative motor deficit was higher compared to patients without M1 infiltration. The mean TTD was 6.8 mm (range 0 mm–30.5 mm). For the short-term motor outcome, all but two patients with motor deterioration had a TTD ≤ 8 mm (Suppl. Fig. 1). The two patients with TTD > 8 mm had a TTD of 10.6 and 10.8 mm and recovered completely over time. No patient with a permanent new motor deficit had a TTD > 8 mm (Suppl. Fig. 1). A tumorous motor cortex infiltration (OR: 4.0, 95 %CI: 1.9–8.3) and a TTD ≤ 8 mm (OR: 4.4, 95 %CI: 2.0–9.7) were associated with higher probability of incomplete tumor resections compared to patients without M1 infiltration or TTD > 8 mm (Table 3).

**Supplementary figure 2:**
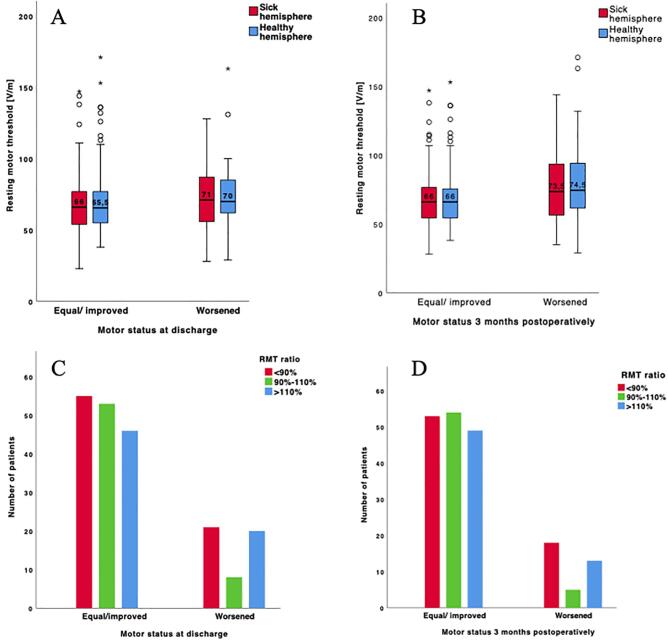


The mean RMT was 71.4 V/m (range: 29–300 V/m; SD: 27.5 V/m) in the healthy and 70.2 V/m (range: 23–293 V/m; SD: 26.3 V/m) in the tumorous hemisphere. Higher RMT_tumor_ and higher RMT_healthy_ values were found in patients with postoperative motor deterioration both on day of discharge and after 3 months (Suppl. Fig. 2).The diffusion analysis revealed a mean FA value of 0.47 (range: 0.22 – 0.62; SD: 0.07) while lower FA values were associated with a higher risk of suffering both a new transient (OR: 3.0, 95 %CI: 1.5–6.0) and a new permanent motor deficit (OR: 7.4, 95 %CI: 2.8–19.5) compared to patients with FA values ≥0.47 (Table 3). Patients with lower FA values had a higher risk of incomplete tumor resection compared to those with FA values ≥0.47 (OR: 4.9, 95 %CI: 2.4–10.1) (Table 3).

**Fig. 2:**
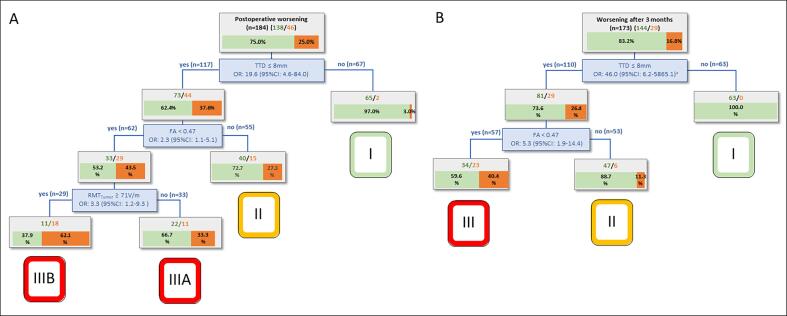
Regression tree analysis for the motor outcome at the day of discharge (A) and after 3 months (B) according to the nTMS risk stratification model (Rosenstock et al., 2021a). Patients with a TTD > 8 mm never suffered from a permanent new motor deficit (risk group I). The FA and the RMT_tumor_ were used to further classify the patients into intermediate risk (group II) and high risk (group III/IIIA and IIIB).

**Fig. 3:**
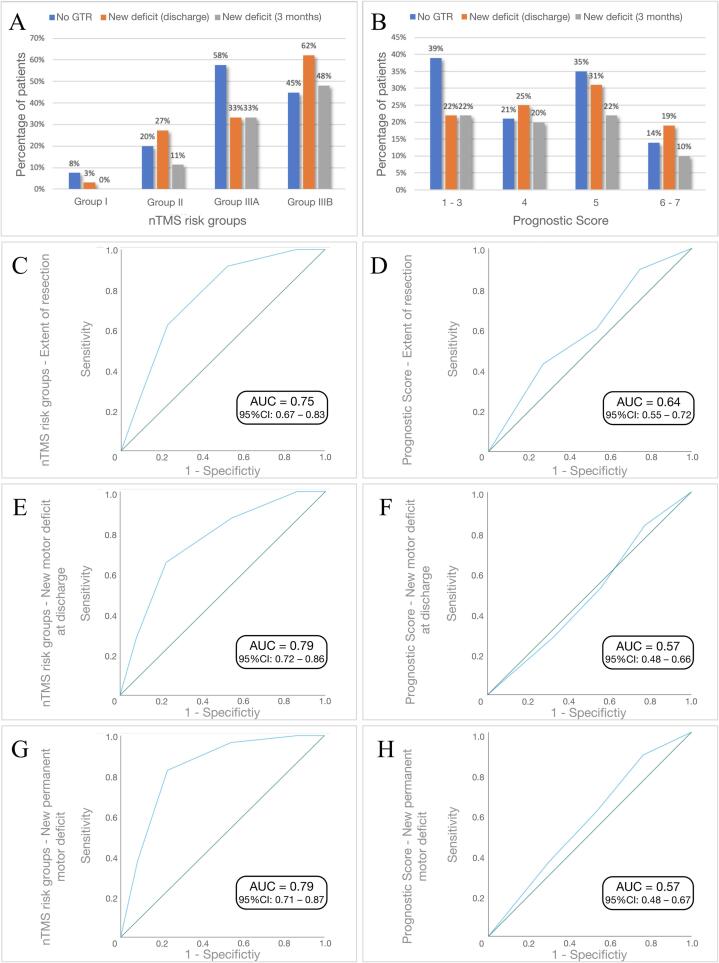
Prediction accuracy of the PrS and nTMS model. Bar charts showing the relative proportions of patients with new transient/new permanent motor deficit and incomplete tumor resection (**A, B**). The prognostic ability of the models was evaluated by calculating ROC curves (C**-H**).

Based on the regression tree analysis of the nTMS model (Fig. 2), patients were divided into risk groups (I-IIIB) stratified by TTD, FA, and RMT_tumor_. In cases with a TTD < 8 mm, the integrity of the CST (measured with the FA value) as well as the cortical exitability (measured with the RMT_tumor_ value) can be used to further stratify the risk for a worsened motor outcome. The nTMS model confirmed the good discrimination ability for a new short-term motor deficit (AUC = 0.79; CI 0.72–0.86) as well as for a new permanent motor deficit (AUC = 0.79; CI 0.71–0.87) (Table 4, Fig. 3).

**Table 4:** Accuracy of the models.

	Postoperative motor outcome					Postoperative motor outcome					EOR					
	(day of discharge)					(after 3 months)										
	Total	Worsening	AUC (95 %CI)	OR (95 %CI)	p	Total	Worsening	AUC (95 %CI)	OR (95 %CI)	p		total	no GTR	AUC (95 %CI)	OR (95 %CI)	p
**nTMS model**											**Combined model**					
**n**	184	46 (25.0%)				173	29 (16.8%)				**n**	184	48 (26.1%)			
I	67	2 (3.0%)	0.79	1	<0.001	63	0	0.79	1	<0.001	I	67	5 (7.5%)		1	
II	55	15 (27.3%)	(0.72–0.86)	12.19 (2.65–56.12)		53	6 (11.3%)	(0.71–0.87)	17.38 (1.97–228.73)		II	55	11 (20.0%)	0.74	1.81 (0.69–4.72)	<0.001
IIIA	33	11 (33.3%)		16.25 (3.34–79.07)		57	23 (40.4%)		86.51 (11.37–11106.94)		IIIA	12	3 (25.0%)	(0.65–0.82)	2.41 (0.55–10.59)	
IIIB	29	18 (62.1%)		53.18 (10.80–261.97)							IIIB	50	29 (58.0%)		9.97 (4.08–24.41)	

**PrS model**																
**n**	203	49 (24.1%)				192	36 (18.8%)									
6-7	43	8 (18.6%)	0.57	1	0.529	41	4 (9.8%)	0.57	1	0.434						
5	48	15 (31.3%)	(0.48–0.66)	1.99 (0.75–5.30)		46	10 (21.7%)	(0.48–0.67)	2.57 (0.74–8.94)							
4	48	12 (25.0%)		1.46 (0.53–4.00)		46	9 (19.6%)		2.25 (0.64–7.96)							
1-3	64	14 (21.9%)		1.23 (0.46–3.23)		59	13 (22.0%)		2.61 (0.79–8.69)							

Patients were divided into classes from I (lowest risk) to IIIB (highest risk for postoperative motor deterioration) according to the regression tree analysis of the nTMS model (Fig. 2). Due to missing FA values in 19 patients, only 184 patients were included in the nTMS model. The subgroups of the PrS model were built according to the original model (Spena et al., 2018). For the extent of resection, an improved/combined model was calculated. **EOR** = Extent of resection. **GTR** = Gross total resection. **AUC** = area under curve. **nTMS** = navigated tanscranial magnetic stimulation. **PrS** = Prognostic sum score.

Although this model was also partially associated with the EOR (AUC = 0.75; CI 0.67–0.83), the RMT_tumor_ (node 3 of the regression tree) does not help in estimating the extent of resection (Fig. 3).

### 3.3 Validation of the prognostic sum score

The parameters of the PrS model and their bivariate association with postoperative motor outcome and EOR are shown in Table 3. No pronounced associations were found between PrS risk parameters and postoperative motor outcome both on day of discharge and after 3 months (Table 3). The presence of a preoperative sensorimotor deficit (OR: 2.0, 95 %CI: 1.1–3.7), a MRI index > 2 (OR: 3.3, 95 %CI: 1.4–7.8) and a tumor volume > 80 ml (OR: 2.3, 95 %CI: 1.0–4.9) were associated with a higher probability of incomplete tumor resection (Table 3) compared to patients without preoperative sensorimotor deficits, a MRI index ≤ 2 or a tumor volume ≤ 80 ml. Contrary to the original PrS model, the presence of a peritumoral cyst was associated with a higher risk for subtotal resections compared to patients with no peritumoral cyst (OR: 2.4, 95 %CI: 1.3–4.8) (Table 3).

Based on the PrS model, subgroups were built according to the model’s specific equation (Table 1): PrS 1–3 (n = 64, 31.5%); PrS 4 (n = 48, 23.6%), PrS 5 (n = 48, 23.6%) and PrS 6–7 (n = 43, 21.2%). The model had low discrimination ability for postoperative (short- or long-term) motor outcome (AUC values below 0.6) but somewhat higher discrimination ability for incomplete tumor resections (Table 4; Fig. 3).

As recurrent or previously operated gliomas were excluded in the original PrS model (Table 1), we performed a subgroup analysis with primary tumors only (n = 149) which did not substantially alter the results (Suppl. data 2).

### 3.4 Combined model to predict the EOR

**Fig. 4:**
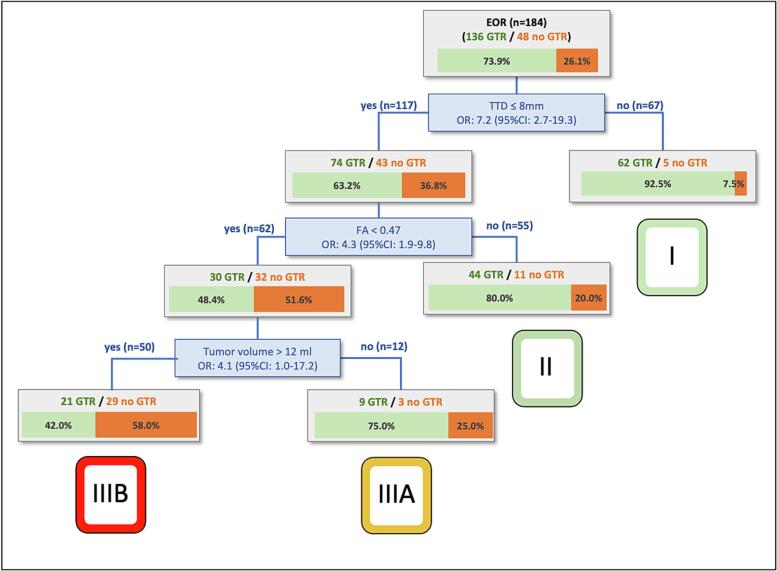
Regression tree analysis for the extent of resection. Parameters of the nTMS model (TTD and FA) and PrS model (tumor volume) were used to classifiy patients into low risk (group I and II), intermediate risk (IIIA) and high risk (IIIB).

To predict the EOR with higher discrimination ability, an improved regression tree model was calculated including all parameters of the nTMS and PrS model that were significantly associated with the EOR(AUC = 0.74; CI 0.65–0.83) (Fig. 4). The calculated regression tree used the TTD and FA from the nTMS model and the tumor volume from the PrS model. Combining the parameters of the nTMS and PrS models that were significantly associated with motor outcome failed to improve either predictive accuracy or discrimination ability for short-term or long-term motor outcome compared to the nTMS model.

## 4 Discussion

### 4.1 Main findings

The validation of the nTMS model on this large prospective data set shows that not only the postoperative motor outcome but also the EOR are associated with nTMS measures. For both, the TTD and the integrity of the CST – measured by the FA value – showed the greatest relevance whereas the cortical excitability – measured by the RMT value – served to further estimate the risk for postoperative motor deterioration. In this cohort, the clinicoradiological PrS score was not substantially associated with the postoperative motor outcome but some parameters of the PrS score were associated with higher probability of incomplete tumor resection. Therefore, a combined model was calculated to predict the EOR more accurately.

### 4.2 Balancing the EOR against the risk of new postoperative motor deficits

In motor-associated (or “eloquent”) tumors, it is always necessary to balance the extent of resection against the risk of new postoperative motor deficits. It is widely known that incomplete tumor resections (STRs) are associated with a higher risk of tumor recurrence and with reduced patient survival (Albuquerque et al., 2021, Brown et al., 2016). Recent studies have explored the concept of supramarginal resection, in which resection was performed beyond the contrast-enhanced or FLAIR-hyperintense tumor portions, but a survival benefit has not yet been demonstrated (Kamp et al., 2019). The nTMS risk model is able to identify cases that (could) benefit from extensive tumor resection and those that are at high risk for new postoperative motor deficits. This is crucial since brain tumor patients with new postoperative neurological deficits experience extensive limitations. In addition to significantly reduced quality of life, the perception of adjuvant therapies is also limited, resulting in reduced survival compared to patients without postoperative neurological deficits (Gulati et al., 2011, Jakola et al., 2011a, McGirt et al., 2009). The risk models studied here aid in preoperative counseling of patients with motor-associated brain tumors (near the motor cortex and/or CST) for a joint risk–benefit consideration to determine the aimed EOR. In selected high-risk cases with recurrent glioma, improved technologies such as interstitial laser thermotherapy (LITT) may provide a less invasive alternative, but surgical resection remains the standard therapy (Chen et al., 2021).

### 4.3 PrS model

The PrS model utilizes 7 clinicoradiological parameters to assign patients with motor-associated brain tumors into 4 risk groups (Spena et al., 2018). Patients at highest risk (PrS score 1–3) had a probability of 92% for incomplete tumor removal and 50% for neurologic deterioration 6 months after surgery. In contrast, a GTR had been achieved in all patients of the best group (PrS score 6–7) without inducing a new motor deficit (Spena et al., 2018). The advantage of the PrS model is its simple and time-efficient applicability, as the contributing variables are determined by the neurological examination and an MRI. However, only association to the outcome after 6 months is included in the PrS model whereas the short-term outcome is not. Furthermore, the MRI index (Spena et al., 2013) and the tumor volume are both parameters representing the relative tumor infiltration, which is thus represented twice in the score. Unfortunately, validation of the score as potential prediction score using the present prospective data set failed for the EOR and the postoperative motor outcome. The EOR was defined by relative measures in the original publication (Table 1) which differs to the present definitions (that are in line with current guidelines (Wykes et al., 2021)) and may explain differences. In addition, the PrS model originally used the modified Rankin scale to assess the postoperative neurological outcome, whereas this dataset was evaluated by the BMRC grading that is more specific for the motor system. Nevertheless, tumor volume as one parameter of the PrS Score was associated with the EOR and improved the prediction model for the EOR. In this cohort, the rate of incomplete tumor resection was twice as high in tumors with peritumoral cyst, which is consistent with the results of other studies (Kiesel et al., 2020, Rosenstock et al., 2022). However, the presence of a peritumoral cyst was associated with an increased likelihood of GTR in the initial PrS model cohort, which might be due to different surgical strategies (e.g., resections of the cyst wall).

### 4.4 nTMS model

The initial nTMS risk model predicts the risk for short- and long-term motor deterioration based on the nTMS-verified motor cortex infiltration, the TTD, and the motor cortex excitability (RMT_ratio_) (Rosenstock et al., 2017b). A bicentric validation (Rosenstock et al., 2021a), including both internal and external validation, but also other studies (Sollmann et al., 2018, Sollmann et al., 2020) confirmed the relevance of these parameters. In addition, the structural integrity of the CST (represented by the DTI-based FA value) was also included in the model as an important risk factor (Rosenstock et al., 2017a). The relevance of the tumorous motor cortex infiltration was reconfirmed, but multivariate regression tree analysis demonstrated a stronger impact of TTD on the postoperative motor outcome, so that the motor cortex infiltration was no longer considered as an independent risk factor in the model. Patients can not only be counseled preoperatively about the risk for a new postoperative motor deficit, but the necessity of the IOM can also be assessed. In a similar patient as the above shown illustrative case, a tumor resection would be offered, but the unfavorable risk constellation for not only transient, but also permanent deficits must be clearly communicated beforehand. As the nTMS-derived values are available preoperatively, the information can be included in the surgical strategy at the respective surgeon’s discretion, e.g. a more conservative threshold for termination of resection during IOM. No parameters of the PrS model were suitable to improve a potential prediction model of the motor outcome. In contrast to short-term motor outcome, an impaired neurophysiological excitability (RMT_tumor_) was not associated to the risk of an incomplete tumor resection. An improved model with superior discrimination ability for the EOR could be calculated by incorporating the tumor volume as one of the relevant parameters of the PrS model. Interestingly, the integrity of the CST – represented by the FA value – was significantly associated with the EOR in addition to the TTD. The tumor volume enables further estimation of the risk of incomplete tumor resection in a subgroup of high risk cases (TTD ≤ 8 mm and FA < 0.47). The association between EOR and the TTD has already been observed in another study (Belotti et al., 2021). Thus, the preoperative assessment indicating the probability and safety of a GTR provides a decision aid for neurosurgeons when counseling patients with motor-eloquent tumors. The use of intraoperative imaging modalities such as intraoperative MRI may be considered in cases with high risk of incomplete tumor resection.

In addition to the discrimination ability, a *meta*-analysis demonstrated that the routine use of preoperative nTMS mapping improves the overall EOR and motor outcome significantly (Raffa et al., 2019). Another advantage is that the preoperative nTMS risk stratification facilitates the IOM (e.g. determining the subcortical stimulation intensity or interpreting heterogeneous phenomena such as a transient decrease of MEP amplitude) (Rosenstock et al., 2021b). In summary, recent studies showed several benefits for nTMS motor mapping and tractography, so patients with motor-eloquent brain tumors should ideally be treated at centers with experience in these techniques (Ille and Krieg, 2021).

### 4.5 Limitations

The objective of this study was to synergistically combine two different models to calculate improved models. The primary outcome parameters of the PrS model differed concerning the definition of the EOR and the clinical outcome (Table 1). In contrast to the nTMS model, no recurrences were included in the PrS model. For this reason, a separate subgroup analysis was performed in Suppl. data 2, which revealed no substantial differences. No recurrences were included in the PrS model, but our subgroup analysis without the recurrences did not show relevant differences (Suppl. data 2). In the present study, we measured absolute residual tumor volumes (as currently recommended (Wykes et al., 2021)) and used the BMRC grading which is more specific for the motor outcome than the mRS. The mRS is a scoring system for the degree of disability and is therefore very important for assessing the patient's ability to perform activities of daily living. In this context, the score is not limited to muscle strength as such, and it is difficult to objectify the extent to which disability is caused by a motor deficit. In contrast, the BMRC score is a classification system for measuring muscle strength within objective categories, but it may not capture other aspects relevant to daily living.

From our point of view, patients with motor-eloquent brain tumors should be treated in large centers that have expertise in the use of nTMS and tractography. Although time and human resources must be allocated for this, the benefit regarding functional outcome and EOR has been confirmed in a *meta*-analysis (Raffa et al., 2019).

The present analysis is retrospective, with known drawbacks. However, a prospective (or even randomized) comparison is not possible for such research questions, because of the low incidence of gliomas in general and for ethical reasons. The combined model predicting the EOR must be validated in further patient populations, since external validity is missing.

## 5 Conclusion

The nTMS model, based on functional and tractography data, was superior to the clinicoradiological PrS model for predicting the motor outcome. An improved/combined model was calculated to estimate the probability for a GTR more accurately. Thus, patient counseling and surgical planning in patients with motor-associated tumors should be performed using functional nTMS data combined with tractography. Prediction of motor outcome/EOR should not be made solely on structural MRI data in these cases.

## Funding

The authors acknowledge the support of the Cluster of Excellence Matters of Activity. Image Space Material funded by the Deutsche Forschungsgemeinschaft (DFG, German Research Foundation) under Germanýs Excellence Strategy – EXC 2025. Dr. Rosenstock is participant in the BIH Charité Digital Clinician Scientist Program funded by the Charité – Universitätsmedizin Berlin, and the Berlin Institute of Health at Charité (BIH). Dr. Belotti received fundings from the Italian Society of Neurosurgery - “Premio Melitta Grasso Tomasello” and the Beretta Foundation for Cancer Study - “European Scholarship on Oncology”.

## CRediT authorship contribution statement

**Meltem Ivren:** Conceptualization, Formal analysis, Investigation, Methodology, Visualization, Writing – original draft. **Ulrike Grittner:** Formal analysis, Methodology, Validation. **Rutvik Khakhar:** Investigation. **Francesco Belotti:** Investigation. **Heike Schneider:** Investigation. **Paul Pöser:** Investigation. **Federico D'Agata:** Methodology. **Giannantonio Spena:** Conceptualization, Methodology, Supervision, Writing – review & editing. **Peter Vajkoczy:** Funding acquisition, Resources, Supervision. **Thomas Picht:** Conceptualization, Funding acquisition, Methodology, Project administration, Resources, Supervision, Writing – review & editing. **Tizian Rosenstock:** Conceptualization, Data curation, Formal analysis, Investigation, Methodology, Project administration, Supervision, Visualization, Writing – original draft.

## Declaration of Competing Interest

The authors declare that they have no known competing financial interests or personal relationships that could have appeared to influence the work reported in this paper.

## Data Availability

The authors do not have permission to share data.
