# Supplementary material for: Comparison of anatomical-based vs. nTMS-based risk stratification model for predicting postoperative motor outcome and extent of resection in brain tumor surgery
Source: Neuroimage Clin. 2023 May 16;38:103436. doi: 10.1016/j.nicl.2023.103436 (PMC10232884; doi:10.1016/j.nicl.2023.103436)
Supplement: Supplementary data 1 [file mmc1.docx]

**Supplement 1. Association between patient characteristics and postoperative motor outcome**

|  | **Postoperative motor outcome**  **(day of discharge)** | | | | **Postoperative motor outcome**  **(after 3 months)** | | | |
| --- | --- | --- | --- | --- | --- | --- | --- | --- |
|  | total | worsening | OR (95%CI) | p | total | worsening | OR (95%CI) | p |
| n | 203 | 49 (24.1%) |  |  | 192 | 36 (18.8%) |  |  |
| **Sex**  Male  Female | 128  75 | 31 (24.2%) 18 (24.0%) | 1.01 (0.52-1.97) 1 | .972 | 119 73 | 24 (20.2%) 12 (16.4%) | 1.28 (0.60-2.76) 1 | .521 |
| **Age in years,** mean (SD) [range] | 50 (15)  [20-81] | 50 (15) [20-81] | 1.03 (0.83-1.27)^a^ | .817 |  | 51(15) [20-81] | 1.05 (0.83-1.34)^a^ | .690 |
| **KPS**  ≤70%  80%  90%  100% | 30  21 72 80 | 8 (26.7%) 5 (23.8%) 13 (18.1%) 23 (28.7%) | 1 0.86 (0.24-3.12) 0.61 (0.22-1.66) 1.11 (0.43-2.85) | .483 | 26 19 68 79 | 6 (23.1%) 6 (31.6%) 12 (17.6%) 12 (15.2%) | 1 1.54 (0.41-5.82) 0.71 (0.24-2.16) 0.60 (0.20-1.79) | .395 |
| **Affected hemisphere** Right  Left  Bilateral | 110 92 1 | 19 (17.3%) 29 (31.5%) 1 (100%) | 1 2.21 (1.14-4.27) | .019 | 102 89 1 | 16 (15.7%) 19 (21.3%) 1 (100%) | 1 1.46 (0.70-3.05) | .315 |
| **Histology** HGG  LGG | 173 30 | 43 (24.9%) 6 (20.0%) | 1.32 (0.51-3.45) 1 | .567 | 163 29 | 31 (19.0%) 5 (17.2%) | 1.13 (0.40-3.19) 1 | .821 |
| **Recurrency** Primary  Recurrence | 149 54 | 32 (21.5%) 18 (31.5%) | 1 1.68 (0.84-3.37) | .143 | 144 48 | 22 (15.3%) 14 (29.2%) | 1 2.28 (1.06-4.93) | .036 |
| **Tumor volume in ml,** mean (IQR) [range] | 26.5  (12.7-53.7) [0.2-224.3] | 26.0  (14.5-40.1) [1.9-147.0] | 0.99 (0.74-1.31)^b^ | .927 | 26.5  (12.4-54.6) [0.2-224.3] | 28.8  (19.1-44.3)  [5.1-224.3] | 1.21 (0.87-1.70)^b^ | .257 |
| **Preop. BMRC grade**  BMRC ≤3  BMRC 4  BMRC 5 | 19 54 130 | 5 (26.3%) 11 (20.4%) 33 (25.4%) | 1 0.72 (0.21-2.42) 0.95 (0.32-2.85) | .750 | 18 49 125 | 2 (11.1%) 11 (22.4%) 23 (18.4%) | 1 2.32 (0.46-11.65) 1.80 (0.39-8.40) | .574 |
| **EOR** GTR  No GTR | 145 58 | 37 (25.5%) 12 (20.7%) | 1 0.76 (0.36-1.59) | .469 | 140 52 | 27 (19.3%) 9 (17.3%) | 1 0.88 (0.38-2.01) | .755 |

**a** – OR calculated for decades in age**. b** – volume was log transformed before analysis. **KPS** = Karnofsky performance status, **HGG** = high grade glioma, **LGG** = low grade glioma, **BMRC** = muscle strength according to the British Medical Research Council, **GTR** = Gross total resection, **SD** = Standard deviation
