# Supplementary material for: Comparison of anatomical-based vs. nTMS-based risk stratification model for predicting postoperative motor outcome and extent of resection in brain tumor surgery
Source: Neuroimage Clin. 2023 May 16;38:103436. doi: 10.1016/j.nicl.2023.103436 (PMC10232884; doi:10.1016/j.nicl.2023.103436)
Supplement: Supplementary data 2 [file mmc2.docx]

**Supplement 4: Association between parameters of nTMS / PrS model with EOR and postoperative motor outcome (subgroup analysis without recurrent gliomas)**

|  | **EOR** | | | | **Postoperative motor outcome**  **(day of discharge)** | | | | **Postoperative motor outcome**  **(after 3 months)** | | | |
| --- | --- | --- | --- | --- | --- | --- | --- | --- | --- | --- | --- | --- |
|  | total | no GTR | OR (95%CI) | p-value | total | worsening | OR (95%CI) | p-value | total | worsening | OR (95%CI) | p-value |
| n | 149 | 40 (26.8%) |  |  | 149 | 32 (21.5%) |  |  | 144 | 22 (15.3%) |  |  |
| **M1 infiltration**  Yes  No | 25 124 | 14 (56.0%) 26 (21.0%) | 4.80 (1.95-11.80) 1 | .001 | 25 124 | 9 (36.0%) 23 (18.5%) | 2.47 (0.97-6.28) 1 | .058 | 24 120 | 7 (29.2%) 15 (12.5%) | 2.88 (1.02-8.10) 1 | .045 |
| **TTD**  ≤ 8mm  > 8mm | 90 59 | 32 (35.6%) 8 (13.6%) | 3.52 (1.49-8.32) 1 | 0.004 | 90 59 | 30 (33.3%) 2 (3.4%) | 14.25 (3.26-62.38) 1 | <.001 | 86 58 | 22 (25.6%) 0 | 39.89 (5.42-508.97) 1 | <.001 |
| **RMT_tumor_** < 71V/m  ≥ 71V/m | 85 64 | 19 (22.4%) 21 (32.8%) | 1.70 (0.82-3.52) 1 | .156 | 85 64 | 16 (18.8%) 16 (25.0%) | 1.44 (0.66-3.15) 16 (25.0%) | .365 | 82 62 | 8 (9.8%) 14 (22.6%) | 2.70 (1.05-6.92) 1 | .039 |
| **RMT ratio**  <90%  90%-110%  >110% | 52 48 49 | 15 (28.8%) 10 (20.8%) 15 (30.6%) | 1.54 (0.61-3.86) 1 1.68 (0.67-4.23) | .514 | 52 48 49 | 13 (25.0%) 5 (10.4%) 14 (28.6%) | 2.87 (0.94-8.78) 1 3.44 (1.13-10.48) | .084 | 49 48 47 | 10 (20.4%) 5 (10.4%) 7 (14.9%) | 2.21 (0.69-7.02) 1 1.51 (0.44-5.13) | .400 |
| **FA** < 0.47  ≥ 0.47 | 52 80 | 19 (36.5%) 12 (15.0%) | 3.26 (1.42-7.51) 1 | .005 | 52 80 | 17 (32.7%) 12 (15.0%) | 2.75 (1.18-6.40) 1 | .019 | 49 78 | 12 (24.5%) 3 (3.8%) | 8.11 (2.16-30.50) 1 | .002 |
| **Tumor margins**  Sharp  Diffuse | 77 72 | 16 (20.8%) 24 (33.3%) | 1 1.91 (0.91-3.98) | .086 | 77 72 | 18 (23.4%) 14 (19.4%) | 1 0.79 (0.36-1.74) | .560 | 74 70 | 11 (14.9%) 11 (15.7%) | 1 1.07 (0.43-2.65) | .887 |
| **Cyst**  Yes  No | 30 119 | 13 (43.3%) 27 (22.7%) | 1 0.384 (0.17-0.89) | .025 | 30 119 | 4 (13.3%) 28 (23.5%) | 1 2.00 (0.64-6.22) | .231 | 30 114 | 5 (16.7%) 17 (14.9%) | 1 0.88 (0.30-2.61) | .812 |
| **Preop. epilepsy**  Yes  No | 77 72 | 18 (23.4%) 22 (30.6%) | 1 1.44 (0.70-2.99) | .324 | 77 72 | 19 (24.7%) 13 (18.1%) | 1 0.67 (0.30-1.49) | .327 | 76 68 | 10 (13.2%) 12 (17.6%) | 1 1.41 (0.57-3.52) | .456 |
| **MRI index**  1-2  3-5 | 34 115 | 4 (11.8%) 36 (31.3%) | 1 3.42 (1.12-10.42) | .031 | 34 115 | 7 (20.6%) 25 (21.7%) | 1 1.07 (0.42-2.75) | .886 | 34 110 | 3 (8.8%) 19 (17.3%) | 1 2.16 (0.60-7.79) | .241 |
| **Tumor volume**  ≤80ml  >80ml | 122 27 | 28 (23.0%) 12 (44.4%) | 1 2.69 (1.13-6.40) | .026 | 122 27 | 27 (22.1%) 5 (18.5%) | 1 0.80 (0.28-2.31) | .680 | 117 27 | 18 (15.4%) 4 (14.8%) | 1 0.96 (0.30-3.10) | .941 |
| **Contrast enhancement** Yes  No | 106 43 | 27 (25.5%) 13 (30.2%) | 0.79 (0.36-1.73) 1 | .553 | 106 43 | 22 (20.8%) 10 (23.3%) | 0.86 (0.37-2.02) 1 | .736 | 103 41 | 18 (17.5%) 4 (9.8%) | 1.96 (0.62-6.19) 1 | .252 |
| **Preop. paresis dysesthesia** Yes  No | 64 85 | 19 (29.7%) 21 (24.7%) | 1.29 (0.62-2.67) 1 | .497 | 64 85 | 13 (20.3%) 19 (22.4%) | 0.89 (0.40-1.96) 1 | 0.764 | 61 83 | 12 (19.7%) 10(12.0%) | 1.79 (0.72-4.46) 1 | .213 |

Testing for group differences was performed with bivariate binary logistic regression. **GTR** = gross total resection. **M1** = motor cortex. **TTD** = tumor-tract distance. **RMT** = resting motor threshold. **FA** = fractional anisotropy.
